# The Multiple Roles of Peptidyl Prolyl Isomerases in Brain Cancer

**DOI:** 10.3390/biom8040112

**Published:** 2018-10-11

**Authors:** Stefano Stifani

**Affiliations:** Department of Neurology and Neurosurgery, Montreal Neurological Institute, McGill University, Montreal, QC H3A2B4, Canada; stefano.stifani@mcgill.ca; Tel.: +1-514-398-3946

**Keywords:** brain cancer, cancer stem-like cells, glioblastoma, medulloblastoma, nuclear factor-kappa B, PIN1, peptidyl prolyl isomerase

## Abstract

Peptidyl prolyl isomerases (PPIases) are broadly expressed enzymes that accelerate the *cis*-*trans* isomerization of proline peptide bonds. The most extensively studied PPIase family member is protein interacting with never in mitosis A1 (PIN1), which isomerizes phosphorylated serine/threonine–proline bonds. By catalyzing this specific *cis*-*trans* isomerization, PIN1 can alter the structure of its target proteins and modulate their activities in a number of different ways. Many proteins are targets of proline-directed phosphorylation and thus PIN1-mediated isomerization of proline bonds represents an important step in the regulation of a variety of cellular mechanisms. Numerous other proteins in addition to PIN1 are endowed with PPIase activity. These include other members of the parvulin family to which PIN1 belongs, such as PIN4, as well as several cyclophilins and FK506-binding proteins. Unlike PIN1, however, these other PPIases do not isomerize phosphorylated serine/threonine–proline bonds and have different substrate specificities. PIN1 and other PPIases are overexpressed in many types of cancer and have been implicated in various oncogenic processes. This review will discuss studies providing evidence for multiple roles of PIN1 and other PPIases in glioblastoma and medulloblastoma, the most frequent adult and pediatric primary brain tumors.

## 1. Peptidyl Prolyl *cis*/*trans* Isomerases

Proline (Pro)-directed serine (Ser)/threonine (Thr) phosphorylation is a widespread mechanism underlying several signaling pathways regulating many cellular processes, such as cell metabolism, proliferation, differentiation, survival, and DNA damage repair, to name only a few. Notable among these pathways are mechanisms regulated by mitogen activated protein kinases (MAPKs) and cyclin-dependent kinases (CDKs), as well as their downstream substrates [1,2]. Protein interacting with never in mitosis A1 (PIN1) is a broadly expressed enzyme that recognizes phosphorylated Ser/Thr (pSer/Thr) residues in pSer/Thr-Pro motifs and catalyzes the *cis*-*trans* isomerization of the pSer/Thr-Pro peptide bond. PIN1-mediated peptidyl prolyl *cis*-*trans* isomerization usually leads to conformational changes in the target protein that can result in modifications of its biological properties, including catalytic activity, stability, protein–protein interaction ability, and binding to DNA or RNA, depending on the particular substrate. Thus, PIN1-mediated *cis*-*trans* isomerization is an important component of many cellular processes in both health and disease through its ability to modulate the activity of phosphorylated proteins involved in key signaling mechanisms (previously reviewed at length in [2,3,4,5,6,7,8]).

PIN1 is the most extensively characterized member of the parvulin subfamily of peptidyl prolyl *cis*-*trans* isomerases (PPIases). In addition to PIN1 and other parvulins, there are two other phylogenetically conserved families of PPIases, termed cyclophilins and FK506-binding proteins (FKBPs). Together, these latter groups of enzymes are referred to as immunophilins due to their shared property of binding to specific immunosuppressive drugs. For instance, in addition to the compound FK506, FKBPs also bind rapamycin, while cyclophilins bind cyclosporine A (reviewed in [9,10,11,12,13,14]). In contrast to PIN1, cyclophilins and FKBPs do not efficiently catalyze the *cis*-*trans* conversion of Ser/Thr-Pro bonds when proline is positioned next to a phosphorylated serine or threonine. Thus, these PPIases do not target pSer/Thr-Pro bonds like PIN1 does. Similar to PIN1, however, members of the cyclophilins and FKBP subfamilies are involved in the regulation of many cellular functions. For example, various cyclophilins and FKBP family members contribute to steroid hormone biology, Alzheimer’s disease pathobiology, virulence, and cardiac function [9,10,11,12,13,14].

Together, PPIase family members act in response to numerous extra- and intracellular cues to regulate the functions of many signaling pathways important for numerous cellular mechanisms. In doing so, these enzymes act as key regulators of multiple biological processes in healthy organisms as well as abnormal processes in pathological conditions ranging from cancer to neurodegeneration to cardiac disease.

## 2. Multiple Roles of PIN1 in Cancer

Dysregulation of proline-directed phosphorylation mechanisms mediated by protein kinases such as MAPKs, CDKs, glycogen synthase kinase 3, and polo-like kinases (PLKs), to name a few, is a common event in cancer [1,2,15,16]. Given the important role played by PIN1 in regulating the functions of substrates of proline-directed phosphorylation, it is therefore not surprising that deregulated PIN1 expression or activity has been associated with numerous cancers (previously reviewed in [17,18,19,20,21]). *PIN1* is aberrantly overexpressed in many tumor cells. For instance, cancers displaying elevated *PIN1* levels include melanoma, breast, prostate, lung, ovarian, oral, and cervical tumors, to name a few [17,18,19,20,21]. Increased *PIN1* expression is usually associated with poorer cancer patient prognosis when compared to non-*PIN1* overexpressors. Notably, elevated *PIN1* levels are correlated with faster recurrence in prostate cancer [18,22]. Similarly, high *PIN1* expression is associated with tumor progression in colorectal and nasopharyngeal cancer [23,24]. 

Several mechanisms have been implicated in aberrant PIN1 expression/activity in cancer. *PIN1* expression is activated downstream of Ras and E2F oncogenes, resulting in PIN1-mediated induction of growth-promoting proteins and inhibitors of differentiation such as β-catenin, c-Jun and NOTCH1, among others [8,17,21]. Loss of tumor suppressors such as BRCA1 is also considered responsible for upregulated PIN1 expression in cancer [25]. Several post-translational mechanisms have also been implicated in the regulation of PIN1 activity. For instance, PLK1 can phosphorylate serine 65 in the catalytic domain of PIN1 to increase its stability. This process is thought to lead to PIN1 overexpression in cancers in which PLK1 is overexpressed [26]. Similarly, MAP3K-related serine-threonine kinase can phosphorylate serine 16 of PIN1, leading to increased cyclin D1 abundance and enhanced tumorigenesis in breast cancer cells [27]. A number of other transcriptional and post-transcriptional mechanisms are also thought to contribute to abnormal PIN1 expression in various cancers (previously reviewed in [17,18,19,20,21]).

The positive correlation between poor clinical prognosis of cancer patients and *PIN1* overexpression is consistent with several biochemical findings. PIN1-mediated peptidyl prolyl *cis*-*trans* isomerization activates, or inactivates, multiple oncogenes and tumor suppressors, respectively. Moreover, PIN1 overexpression causes chromosome instability [17,18,19,20,21,28]. Conversely, *PIN1*-null mice are resistant to breast cancer induced by overexpression of oncogenes [29,30,31]. Consistently, PIN1 impairment suppresses Neu- and Ras-induced transformation of mammary epithelial cells through activation of cyclin D1 [32]. Similarly, inhibition of PIN1 impairs cancer progression in a mouse model of NOTCH3-induced T-cell acute lymphoblastic leukemia [33]. Several additional mechanisms are believed to underlie the involvement of PIN1 in the development of multiple cancers, including the regulation of numerous transcription factors responsive to growth-inducing signals. These and other processes have been reviewed previously (e.g., [17,18,19,20,21,22]) and thus will not be addressed in detail here.

Together, these observations point to important roles of PIN1 in oncogenic pathways. This review will focus on the involvement of PIN1, as well as other PPIases, in glioblastoma and medulloblastoma, the most frequent adult and pediatric primary brain tumors, respectively.

## 3. Involvement of PIN1 in Glioblastoma

Gliomas are the most common primary brain tumors. They comprise low-grade infiltrative astrocytomas (World Health Organization grade II) and high-grade tumors (grades III and IV). Grade IV glioma, referred to as glioblastoma, is characterized by aggressive growth, extensive invasion of brain tissue, and almost inevitable recurrence. Glioblastoma is virtually untreatable, even after combined surgery, radiation therapy and chemotherapy, and glioblastoma patients have an average survival of less than two years [34,35,36]. 

Glioblastoma is a highly heterogeneous cancer exhibiting a build-up of a variety of poorly differentiated neural cells that include, at a minimum, two main groups of cancer cells, operationally referred to as resting and proliferating glioblastoma cell pools. The resting cell population is hypothesized to be rare and comprised of quiescent cells that have undergone reversible exit from the cell cycle (capable of re-entering mitosis if activated by specific stimuli) and stem-like cells thought to be slowly cycling cells endowed with self-renewing potential and capable of generating rapidly amplifying progeny cells. The proliferating glioblastoma cell population is comprised of a collection of more prevalent cells undergoing rapid proliferation and exhibiting various degrees of incomplete differentiation. The cellular heterogeneity of glioblastoma makes treatment extremely challenging because different cancer cells within the same tumor may not respond equally to treatment. Current therapies usually target the proliferating cell pool, which is considered as the main contributor to processes underlying glioblastoma development, such as tumor growth and invasion. Unfortunately, these approaches have failed to treat glioblastoma, suggesting that stem-like and/or quiescent cell pools are not targeted by current therapies and may represent the population(s) responsible for glioblastoma therapy resistance and recurrence after surgery and treatment [36,37,38]. These observations underscore the importance of gaining a better understanding of the pathobiology of both the resting and proliferating glioblastoma cell pools and how these cells contribute to gliomagenesis. 

*PIN1* is overexpressed in human gliomas compared to non-cancerous brain cells [39,40,41]. Importantly, the relative levels of *PIN1* expression are positively correlated with enhanced brain tumor progression [41]. Although the mechanisms responsible for PIN1 upregulation in gliomas remain to be fully defined, several studies provide evidence for multiple PIN1 functions in glioblastoma. 

### 3.1. Role of PIN1 in Gliobastoma Cell Survival

One of the first suggestions that PIN1 might contribute to gliomagenesis came from the demonstration that PIN1 binds to death-associated protein DAXX in glioblastoma cell lines. This interaction occurs when DAXX is phosphorylated at serine(178)-proline and results in increased degradation of DAXX via the ubiquitin-proteasome pathway, thereby interfering with DAXX-mediated cellular apoptosis. Consistent with this effect, RNA interference (RNAi)-mediated inhibition of PIN1 in A172 glioblastoma cells enhances cell death responses and is accompanied by increased DAXX induction and activation of apoptotic pathways [39]. These findings provided the first evidence suggesting the existence of PIN1-mediated anti-apoptotic mechanisms in glioblastoma. This possibility is consistent with the results of later studies in which endogenous PIN1 activity was inhibited in glioma cells using the potent PIN1 pharmacological inhibitor, juglone. Treatment of glioblastoma cells with juglone significantly decreases cell proliferation and induces apoptosis, as well as caspase-3 activity, in a dose-dependent manner [42,43]. These findings suggest that PIN1 is involved in mechanisms promoting the survival of glioblastoma cells by inhibiting apoptosis. This possibility is in accordance with the demonstration that PIN1 is involved in regulating various aspects of apoptosis in different biological contexts, including a variety of cancer cells. For instance, PIN1 can promote the oncogenic functions of activated NOTCH and mutant p53 in breast cancer cells, leading to deregulated mitochondrial pro-survival mechanisms [44,45]. 

### 3.2. Involvement of PIN1 in Glioblastoma Cell Migration

It was through the combined use of *PIN1*-directed RNAi reagents and juglone-mediated pharmacological inhibition that PIN1 impairment was shown to also impact the migratory potential of glioblastoma cells, resulting in decreased cell migration in vitro [42]. Participation of PIN1 in molecular pathways regulating cancer cell migration is in accordance with the identification of PIN1 substrates with an involvement in cell motility. Notably, PIN1 was recognized as a modulator of RelA-containing nuclear factor-kappa B (NF-κB) dimers by binding to RelA phosphorylated at threonine(254)-proline, accelerating *cis*-*trans* isomerization at this site, and leading to RelA stabilization and increased transcription of NF-κB target genes, thereby promoting NF-κB signaling [46]. This function is relevant in the context of glioblastoma because PIN1 can enhance NF-κB activity in glioblastoma cells [40]. NF-κB signaling promotes glioblastoma cell invasion by transactivating the expression of several genes encoding molecules promoting cell motility, including fibroblast growth factor inducible 14, a member of the tumor necrosis factor (TNF) receptor superfamily, interleukin-8 (IL-8), monocyte chemoattractant protein 1, and CXC chemokine receptor 4, to name a few [47,48]. Together, these observations suggest that PIN1-mediated mechanisms are important for cancer cell invasion in glioblastoma, a possibility also consistent with a role of PIN1 in supporting cell invasion in breast cancer [49].

### 3.3. Role of PIN1 in Glioblastoma Angiogenesis 

PIN1-mediated mechanisms have also been implicated in the promotion of angiogenesis in glioblastoma. PIN1 impairment was proposed to affect the angiogenic potential of glioblastoma cell lines based on chick chorioallantoic membrane assays [42]. Consistently, PIN1 attenuation leads to decreased levels of vascular endothelial growth factor (VEGF) and matrix metalloproteinase 9 in glioma cells [43]. This finding is in accordance with the ability of PIN1 to promote *VEGF* expression, and a consequent enhancement of angiogenesis, during breast cancer progression [50]. PIN1 promotes the activity of other factors with the potential to promote angiogenesis in glioblastoma, in addition to VEGF. As mentioned above, PIN1 activates RelA-containing NF-κB dimers. NF-κB activates the expression of *VEGF* and *IL-8*, another pro-angiogenic gene, in glioma cells [51,52]. Moreover, NF-κB impairment significantly reduces glioblastoma growth and angiogenesis in nude mice [52]. These combined results provide evidence suggesting that PIN1 overexpression is correlated with mechanisms promoting angiogenesis in glioblastoma including, but not limited to, the promotion of NF-κB, VEGF, and IL-8 activation. In agreement with this interpretation, PIN1 was shown to contribute to angiogenesis in colon cancer cells by interacting with hypoxia-inducible factor (HIF)-1α in a phosphorylation-dependent manner, leading to HIF-1α stabilization and increased transcriptional activation of *VEGF* [53]. 

### 3.4. Participation of PIN1 in Warburg Effet in Glioblastoma

The Warburg effect is a mechanism contributing to tumor progression in many cancers. It consists in the choice by the tumor cells to mainly produce energy by glycolysis in the cytosol, followed by lactic acid fermentation, rather than by pyruvate oxidation in mitochondria [54,55]. The mechanisms underlying the coordinated enhancement of cytosolyic glycolysis and suppressed mitochondrial pyruvate metabolism in the Warburg effect are incompletely elucidated, but recent studies by Lu and colleagues have implicated PIN1 in these mechanisms [56,57]. Specifically, it was shown that epidermal growth factor receptor (EGFR)-activated ERK2 binds to pyruvate kinase M2 (PKM2), the enzyme that catalyzes the last step of glycolysis, namely dephosphorylation of phosphoenolpyruvate to pyruvate. ERK2 phosphorylates PKM2 at Ser(37)-Pro in glioblastoma cells. This event leads to recruitment of PIN1 to PKM2 and *cis*-*trans* isomerization of the pSer(37)-Pro bond, promoting PKM2 translocation to the nucleus, where PKM2 promotes carcinogenesis at least in part by inducing c-MYC expression [56]. In subsequent studies, PIN1 was shown to also play an important role in the regulation of phosphoglycerate kinase 1 (PGK1), a key enzyme in the glycolytic pathway that catalyzes the generation of adenosine triphosphate (ATP) and 3-phosphoglycerate from adenosine diphosphate (ADP) and 1,3-diphosphoglycerate. PGK1 is upregulated in several cancers, including breast, pancreatic, ovarian, and brain cancer [54,55]. PIN1 binds to PGK1 phosphorylated at Ser(203)-Pro in response to ERK activation in glioblastoma cells. The ensuing *cis*-*trans* isomerization of this peptide bond by PIN1 leads to translocation of PGK1 to mitochondria, where PGK1 phosphorylates and activates pyruvate dehydrogenase kinase 1, which in turn phosphorylates and inhibits the pyruvate dehydrogenase complex responsible for transforming pyruvate into acetyl-CoA. This action results in reduced mitochondrial pyruvate use, altered reactive oxygen species (ROS) production, and increased lactate synthesis, leading to promotion of gliomagenesis [57]. Together, these findings provide evidence for roles of PIN1 in promoting the Warburg effect in glioblastoma cells, revealing an addition mode of PIN1 involvement in this deadly brain cancer.

### 3.5. Open Questions

The involvement of PIN1 in multiple oncogenic processes in glioblastoma suggests that PIN1-mediated *cis*-*trans* isomerization mechanisms contribute to gliomagenesis and that strategies aimed at impairing PIN1 activity might be attractive anti-glioma therapies. Consistent with this possibility, *PIN1* single nucleotide polymorphisms causing decreased PIN1 expression are associated with a reduced risk for multiple cancers [58]. A number of considerations must be kept in mind, however. Most previous studies of PIN1 involvement in glioblastoma suffer from a common limitation, namely the fact that they were mainly based on in vitro investigations using established glioblastoma cell lines. These cell culture systems can hardly recapitulate the in vivo heterogeneity of glioblastoma, making it virtually impossible to discern in which specific glioblastoma cell subpopulations PIN1 exerts its main functions. A case in point is offered by the suggestion that abnormal PIN1 activation enhances glioblastoma cell survival through inhibition of apoptotic pathways. It remains to be determined whether PIN1 mediates a pro-survival effect in the proliferating or the resting glioblastoma cell pool (or both) in vivo. Another example is provided by the hypothesized role of PIN1 in promoting cancer cell migration. Although it is possible that this function might occur because of an increase in the fraction of more developmentally advanced glioblastoma cells resembling the migratory neural cells in the healthy brain (e.g., oligodendrocyte and/or astrocyte precursor cells), the lack of information on the cancer cell subtypes in which PIN1 is specifically overactivated leaves the door open to other interpretations, including the possibility that PIN1 might increase the migratory potential of most glioblastoma cell types. To address these, as well as other, open questions it will be necessary to precisely characterize the features of the specific glioblastoma cells in which PIN1 is aberrantly activated.

The identity of most of the specific PIN1 substrates that are impacted by PIN1 overexpression/overeactivation in ways contributing to gliomagenesis also remains to be determined. Given the long list of oncogenes and tumor suppressors that are known to be targets of PIN1, it is reasonable to suggest that PIN1 has the potential to participate in multiple oncogenic processes in glioblastoma through the modulation of a broad range of cellular processes, including but not limited to cell proliferation, survival, motility, DNA repair, angiogenesis, and metabolism. It will be necessary to increase our understanding of the downstream targets of PIN1 during specific oncogenic mechanisms, and in distinct glioblastoma cell populations, to be able to further elucidate the contribution of PIN1 to gliomagenesis. 

## 4. Roles of Other Peptidyl Prolyl Isomerases in Glioblastoma

### 4.1. Cyclophilins

The first suggestion that at least some members of the cyclophilin family might be involved in gliomagenesis came from the observation that expression of cyclophilin A (CypA) is elevated in human glioblastoma cell lines and tissues when compared to normal astrocytes and control brain tissue [59,60]. Han and colleagues correlated this observation with the demonstration that two CypA-directed immunosuppressive drugs, cyclosporine A and sanglifehrin A, enhance apoptosis induced by the anti-cancer chemotherapy drug cisplatin in C6 glioma cells [59]. This enhancement of apoptotic cell death is associated with an increase in ROS generation and a decrease in intracellular glutathione levels. In agreement with these observations, cisplatin-induced apoptosis can also be enhanced by RNAi-mediated CypA knockdown. In subsequent studies, Sun and colleagues showed further that attenuation of CypA in glioma cells causes decreased proliferation, migration and anchorage-independent growth in vitro. More importantly, this manipulation also causes slower growth of brain tumor xenografts initiated by CypA-attenuated cells in vivo [60]. Cyclophylin A knockdown was shown to result in reduced NF-κB activation and reduced expression of the NF-κB target gene, *IL-8* [60]. These findings are similar to the impact of PIN1 attenuation on NF-κB activation, as described above, and suggest further that PPIase-mediated modulation of NF-κB signaling may represent an important mechanism of gliomagenesis. In more recent studies, Wang and colleagues asked whether CypA might be involved in glioblastoma stem-like cell (GSC) pathobiology. Using glioblastoma patient derived GSC cultures, these authors provided evidence that CypA promotes GSC self-renewal, proliferation, and radiotherapy resistance. Moreover, these studies showed that CypA can bind β-catenin and increase the interaction between β-catenin and transcription factor-4 (TCF4), thereby enhancing wingless-related integration site WNT target gene activation in GSC cultures [61]. 

Similar to PIN1 and CypA, cyclophilin B (CypB), a PPIase residing in the endoplasmic reticulum, is also upregulated in malignant gliomas. CypB is important for glioma cell proliferation and survival as shown by the impact of its attenuation, as well as inhibition using cyclosporine A, in cultured U251 glioblastoma cells [62]. In agreement with these phenotypes, impairment of CypB results in induction of cellular senescence, cell death from loss of MYC and mutant p53, and Janus-activated kinase/signal transducer and activator of transcription-3 (STAT3) signaling. Together, these findings provide evidence suggesting that CypB supports MYC and p53-dependent cell survival in glioblastoma and contributes to sustained expression of oncogenic proteins [62]. Importantly, the same studies also showed the presence of increased ROS, expansion of the endoplasmic reticulum, and abnormal unfolded protein responses in CypB-depleted glioblastoma cells [62]. Consistent with these observations, recent work has shown that inhibition of cyclophilins in glioblastoma cells induces parapoptosis, a type of cell death morphologically distinct from apoptosis and necrosis, at least in part as consequence of buildup of unfolded/misfolded proteins in the endoplasmic reticulum [63].

### 4.2. FK506-Binding Proteins

Studies of FKBP subfamily members have uncovered additional, and in some case alternative, functions for PPIases in glioblastoma. Notably, the 52-kDa FK506-binding protein (FKBP52) is involved in mechanisms that suppress tryptophan-2,3-dioxygenase (TDO)-mediated tryptophan catabolism in glioblastoma [64]. Tryptophan catabolism is an important contributor toward an immune tolerant (tolerogenic) microenvironment in glioblastoma and other cancers [64]. Increased metabolism of tryptophan has the deleterious consequence of perturbing the balance of regulatory and effector responses of the immune system, creating a tolerogenic environment that allows evasion of host immune responses by cancer cells [65]. Glioblastoma has the ability to exploit tryptophan catabolism-mediated tolerogenic mechanisms at least in part though TDO upregulation [64]. FKBP52 is a regulator of steroid hormone receptor signaling and has been implicated in a variety of hormone-dependent cancers [13]. In the context of glioblastoma, FKBP52 cooperates with the glucocorticoid receptor to inhibit TDO expression. Consistently, FKBP52 knockdown or inhibition with FK506 leads to increased TDO activity in glioblastoma cells [64]. These findings provide evidence for a role of FKBP52 in steroid-responsive anti-glioma mechanisms targeting TDO, a rate-limiting enzyme in tryptophan catabolism. 

FK506-binding protein 38 (FKBP38) is a chaperone that is preferentially localized to mitochondria and differs from other PPIases because its enzymatic activity is regulated by the calcium sensor calmodulin [66]. FKBP38 was shown to inhibit apoptosis, at least in part by recruiting the anti-apoptotic proteins Bcl-2 and Bcl-xL to mitochondria [66,67]. Pistollato and colleagues showed that FKBP38 activity is inhibited by BMP2 in glioblastoma cells [68]. This effect is thought to be part of a mechanism through which BMP2 promotes cancer cell differentiation by impairing the stability of hypoxia inducible factor one alpha (HIF-1α) activated in response to hypoxia. More specifically, bone morphogenetic protein-2 (BMP2) promotes HIF-1α downregulation by inhibiting FKBP38-mediated degradation of proline hydroxylase 2, which is involved in HIF-1α proline hydroxylation and ensuing proteasomal degradation [68]. Thus, elevated FKBP38 levels are associated with increased HIF-1α activity and tumor progression in glioblastoma as part of mechanisms that de-sensitize cancer cells to pro-differentiating BMP2 signals.

Together, these studies provide evidence for multiple roles of cyclophilins and FKBPs in glioblastoma and suggest that drugs that would selectively inhibit their PPIase activity without causing immune suppression might represent attractive candidates for anti-glioma therapy.

### 4.3. Other Parvulins

Parvulin 14 (Par14) is encoded by the gene *PIN4*, a parvulin family PPIase related to PIN1 [69]. *PIN4* also encodes a longer PPIase, termed Par17, as a result of alternative transcription initiation [69,70]. Unlike PIN1, Par14 does not accelerate the *cis*-*trans* interconversion of pSer/Thr-Pro bonds and instead shows preference for arginine–proline moieties [69,70]. A number of functions have been associated with Par14, including regulation of cell cycle progression, metabolic pathways and ribosome biogenesis [70].

Recent studies by Frattini and colleagues have shown that tyrosine-122 (Tyr-122) of Par14 (referred to as PIN4 in their studies) is phosphorylated in a subset of glioblastoma cases expressing the oncogenic fusion protein FGFR3-TACC3 (F3-T3) [71]. Using phospho-PIN4(Tyr122)-specific antibodies, these authors showed that F3-T3-positive tumors express higher levels of phospho-PIN4(Tyr122) compared to tumors lacking this fusion protein. More importantly, a series of elegant investigations showed that phosphorylation of PIN4 at Tyr-122 in response to F3-T3 establishes a “F3–T3–PIN4” axis that triggers biogenesis of peroxisomes and synthesis of new proteins. Activation of these pathways leads to the accumulation of intracellular ROS, increasing mitochondrial respiration and tumor growth. Thus, the phosphorylation of PIN4 at Tyr-122 is an important intermediate step in F3-T3-induced activation of oxidative phosphorylation and mitochondrial biogenesis in glioblastoma cells [71]. These important recent findings provide evidence further implicating PPIase family members in the control of metabolic functions in glioblastoma.

Taken together, these findings show that several PPIases, in addition to PIN1, participate in the regulation of glioblastoma growth, survival, angiogenesis, and metabolism, thus providing further evidence for multiple functions of these enzymes in gliomagenesis. Notably, a number of different studies are providing growing evidence for critical roles for PPIases in glioblastoma cell metabolism, including promoting the Warburg effect, regulating tryptophan catabolism, and increasing mitochondrial respiration in response to the expression of oncogenic fusion proteins. Previous studies also suggest, however, that different PPIases might have different functions in glioblastoma, underscoring the importance of continued investigations into the roles of several PPIase family members in this context.

## 5. Involvement of PIN1 in Medulloblastoma

Medulloblastoma, a highly invasive neuroectodermal tumor of the cerebellum, is the most common malignant brain tumor in childhood. It can also occur in adolescents and even young adults [72]. Like glioblastoma, medulloblastoma is also highly heterogeneous. At least five subtypes were originally described based on histopathological criteria: classic, desmoplastic, anaplastic, large cell, and medulloblastoma with extensive nodularity [73,74]. Subsequent genetic and molecular analyses revealed that mutations in the Sonic hedgehog (SHH) pathway are common in desmosplastic and extensively nodular medulloblastoma. Alterations in WNT signaling are mainly associated with classic medulloblastoma, whereas *MYC* or *MYCN* amplifications are found in large cell/anaplastic variants. Based on these and other observations, medulloblastoma is now considered as comprised of at least four main subgroups with distinctive molecular, demographic and clinical characteristics, termed SHH, WNT, group 3 and group 4 medulloblastoma variants [72,73,74,75,76,77,78]. 

The SHH subgroup corresponds to about 30% of medulloblastoma cases and usually has a less severe prognosis than other subtypes, especially group 3. The WNT subgroup is relatively rare (<10% of cases) and has a better prognosis compared to the other groups. Group 3 tumors are often characterized by elevated MYC expression, account for approximately 25% of medulloblastoma cases, and are associated with the worst overall survival among all subtypes. Group 4 tumors are the most frequent medulloblastomas (~35% of cases), have a heterogeneous genetic makeup, and are associated with an intermediate prognosis [75,76,77,78,79,80]. The complexity and heterogeneity of medulloblastoma calls for an improved understanding of the molecular and cellular mechanisms underlying the development of different medulloblastoma subtypes.

Recent work has provided evidence that implicates PIN1 in SHH-medulloblastoma. In canonical SHH signaling, binding of SHH to a membrane-bound Patched (PTCH) receptor, such as PTCH1, leads to depression of Smoothened (SMO), a membrane-bound G-protein–coupled receptor-like protein, which then promotes the translocation of activated glioma-associated oncogene homolog (GLI) transcription factors (e.g., GLI1) into the nucleus. GLI transcription factors then activate the expression of SHH target genes such as *CCND1* (encoding cyclin D1) and *MYCN*, among others [81]. A search for GLI1-binding proteins in medulloblastoma showed that GLI1 and PIN1 form a physical complex in MED-311FH medulloblastoma cells and that this interaction leads to increased GLI1 protein abundance in vitro [82]. Based on this finding, Xu and colleagues investigated the consequence of PIN1 inhibition on SHH-medulloblastoma tumorigenesis in vivo. Using a genetically engineered SMO-induced mouse model of SHH-medulloblastoma, these authors demonstrated that *PIN1* knockout in these mice has no detectable effect on normal cerebellar development but impairs tumor development and increases survival. Moreover, the expression of SHH target genes, like *GLI1*, *GLI2*, *PTCH2*, and *forkhead box A2* (*FOXA2*), is significantly reduced in the cerebellum of transgenic mice in which *PIN1* was inactivated [82]. These findings raise the possibility that PIN1 contributes to the development of SHH-driven medulloblastoma, at least in part by enhancing GLI1 activity upon direct binding to phosphorylated GLI1. In turn, this possibility suggests that mechanisms involving interaction between GLI1 and PIN1 may represent potential therapeutic targets in SHH-driven medulloblastoma tumorigenesis.

These recent findings raise a number of important questions. The normal roles of PIN1 during cerebellar development, and the physiological significance of its ability to interact with GLI1 in this context, remain to be fully defined. Addressing these questions will require a better characterization of the impact of PIN1-mediated isomerization on GLI1-regulated mechanisms, as well as a better understanding of the main substrates of PIN1 in cerebellar cells. These substrates may include other drivers of SHH-medulloblastoma tumorigenesis, in addition to GLI1. Furthermore, although the absence of noticeable deleterious effects as a result of *PIN1* loss on cerebellar development in mice suggests that selective PIN1 inhibitors could hold promise for medulloblastoma therapeutic strategies, the possibility of affecting pathways controlling other cellular mechanisms, in addition to cell survival, needs to be carefully considered in this context.

## 6. Targeting Peptidyl Prolyl Isomerases for Brain Cancer Treatment

The study of PIN1 and other PPIases in numerous cancers, including brain tumors, has revealed a number of important functions for these enzymes in oncogenic mechanisms. This situation is not surprising when PIN1 is considered, given the key role played by PIN1 in the modulation of numerous oncogenes and tumor suppressors phosphorylated at pSer/Thr-Pro sites, and the fact that proline-directed phosphorylation and dephosphorylation mechanisms are shared by numerous oncogenic pathways in many tumor cells. These observations suggest that PIN1 might be a potentially attractive target of cancer therapies aimed at attenuating/impairing PIN1 functions in ways that would impact on a number of oncogenic mechanisms. As already mentioned above, this possibility is supported by the previous demonstration that *PIN1* single nucleotide polymorphisms resulting in decreased PIN1 expression are associated with reduced cancer risk [58]. Consistent with the pleiotropic functions of PIN1, there is growing evidence that PIN1 might play roles in both less abundant cancer stem-like cells and more prevalent proliferating cells in various tumors [17,18,19,20,21]. Moreover, several PIN1-activated proteins are currently difficult to target pharmacologically and the opportunity to inhibit PIN1 might provide an indirect way to regulate at least some of them. Together, these observations suggest that PIN1 inhibition, in combination with other pharmacological approaches, might have effects on various cancer cell subtypes, possibly including those believed to be particularly resistant to current therapies. In the context of glioblastoma, it should again be emphasized that the limited information about the identity of the glioblastoma cell types in which PIN1 activity is deregulated, as well as the molecular substrates of PIN1 in these cells, represents a challenge that needs to be addressed before PIN1-mediated mechanisms can be viewed as potentially attractive targets of anti-glioma therapies.

PIN1 has also received increasing attention in the cancer field because potent PIN1 pharmacological inhibitors already exist and some of them, i.e., juglone, have already been shown to inhibit tumor cell proliferation and survival. PIN1 inhibitors might have limited toxicity, while at the same time impacting on numerous cancer cell subtypes, given the fact that *PIN1*-null mice have relatively few phenotypes and develop to adulthood [4,9,20,21,22]. In the specific case of potential applications to brain cancer treatment, however, it is worth mentioning that PIN1 is important for neuronal development [83] and its dysfunction is associated with neuronal degeneration and Alzheimer’s disease pathophysiology [84,85]. Although the potential risk of PIN1-directed therapy-associated neurodegeneration could raise concern, it is likely that no side effects would emerge during short-term cancer treatments with PIN1 inhibitors. Similarly, drugs that would selectively inhibit cyclophilin and/or FKBP activity without immune suppression might hold potential for treatment of several cancers, including glioblastoma [14,55]. Targeting PIN4 might also have potential in glioblastoma, as suggested by the recent implication of PIN4 in F3-T3-driven gliomagenesis in at least a subset of glioblastoma cases [71]. 

As mentioned, a number of factors need to be carefully considered before the use of PPIase inhibitors in glioblastoma and/or medulloblastoma therapies is pursued further. Given the demonstrated involvement of PPIase-mediated mechanisms in the regulation of multiple molecular mechanisms relevant to brain cancer pathobiology, such as cell proliferation, survival, motility, metabolism, and angiogenesis, it is likely that these enzymes contribute to carcinogenesis in a number of ways and by acting on a variety of different substrates. Given our still limited understanding of PPIase roles in brain cancers, pharmacological approaches targeting specific PPIases will have to be carefully assessed with regards to their impact on multiple signaling mechanisms and on different brain cancer cell subtypes. In the case of glioblastoma, for example, it is possible that these enzymes may be differentially activated in different cancer cell subpopulations (i.e., glioma stem-like cells vs. more developmentally advanced cancer cells) and thus the effect of PPIases inhibition may cause changes in the relative distribution of different cell pools that could result in unanticipated effects. 

## 7. Concluding Comments

Abnormal activation of PIN1 and other PPIases is frequently observed in brain tumors. A better understanding of the variety of functions played by PPIases in glioblastoma and medulloblastoma, especially the identification of the oncogenes and tumor suppressors whose activity is regulated by these enzymes, might lead to important new insight into the pathobiology of these deadly cancers. Moreover, the characterization of the effect of *cis*-*trans* isomerization on the activities of the targets of PIN1 and other PPIases, in part through the development of conformation-specific antibodies, might contribute to a better mechanistic and diagnostic understanding of these tumors. The promising findings already described using PPIase-directed inhibitors is raising hope that targeting these molecules may provide means to simultaneously inhibit several oncogenic mechanisms, with limited anticipated toxicity. The development of new and more selective inhibitors of PIN1 and other PPIases is therefore considered as an important step for anti-cancer approaches in several tumors, including brain cancers.
